# A systematic literature review of the disease burden in patients with recessive dystrophic epidermolysis bullosa

**DOI:** 10.1186/s13023-021-01811-7

**Published:** 2021-04-13

**Authors:** Jean Yuh Tang, M. Peter Marinkovich, Eleanor Lucas, Emily Gorell, Albert Chiou, Ying Lu, Jodie Gillon, Dipen Patel, Dan Rudin

**Affiliations:** 1 Department of Dermatology, Stanford Universixsty School of Medicine, 291 Campus Drive, Stanford, CA 94305 USA; 2 Pharmerit – An OPEN Health Company, 4350 East West Highway, Suite 1100, Bethesda, MD 20814 USA; 3 Abeona Therapeutics Inc, 1330 Avenue of the Americas, New York, NY 10019 USA

**Keywords:** Recessive dystrophic epidermolysis bullosa, Burden of disease, Systematic literature review

## Abstract

**Background/objective:**

Recessive dystrophic epidermolysis bullosa (RDEB) is a genetic collagen disorder characterized by skin fragility leading to blistering, wounds, and scarring. There are currently no approved curative therapies. The objective of this manuscript is to provide a comprehensive literature review of the disease burden caused by RDEB.

**Methods:**

A systematic literature review was conducted in MEDLINE and Embase in accordance with PRISMA guidelines. Observational and interventional studies on the economic, clinical, or humanistic burden of RDEB were included.

**Results:**

Sixty-five studies were included in the review. Patients had considerable wound burden, with 60% reporting wounds covering more than 30% of their body. Increases in pain and itch were seen with larger wound size. Chronic wounds were larger and more painful than recurrent wounds. Commonly reported symptoms and complications included lesions and blistering, anemia, nail dystrophy and loss, milia, infections, musculoskeletal contractures, strictures or stenoses, constipation, malnutrition/nutritional problems, pseudosyndactyly, ocular manifestations, and dental caries. Many patients underwent esophageal dilation (29–74%; median dilations, 2–6) and gastrostomy tube placement (8–58%). In the severely affected population, risk of squamous cell carcinoma (SCC) was 76% and mortality from SCC reached 84% by age 40. Patients with RDEB experienced worsened quality of life (QOL), decreased functioning and social activities, and increased pain and itch when compared to other EB subtypes, other skin diseases, and the general population. Families of patients reported experiencing high rates of burden including financial burden (50–54%) and negative impact on private life (79%). Direct medical costs were high, though reported in few studies; annual payer-borne total medical costs in Ireland were $84,534 and annual patient-borne medical costs in Korea were $7392. Estimated annual US costs for wound dressings ranged from $4000 to $245,000. Patients spent considerable time changing dressings: often daily (13–54% of patients) with up to three hours per change (15–40%).

**Conclusion:**

Patients with RDEB and their families/caregivers experience significant economic, humanistic, and clinical burden. Further research is needed to better understand the costs of disease, how the burden of disease changes over the patient lifetime and to better characterize QOL impact, and how RDEB compares with other chronic, debilitating disorders.

**Supplementary Information:**

The online version contains supplementary material available at 10.1186/s13023-021-01811-7.

## Introduction

Recessive dystrophic epidermolysis bullosa (RDEB) is a rare, severe form of dystrophic epidermolysis bullosa (DEB), a genetic collagen disorder characterized by skin fragility and scarring of the skin from birth onwards. Patients with this disorder inherit mutations in both alleles of *COL7A1*, the gene which produces type VII collagen (C7). This leads to absences or irregularities in C7 and alterations in the character and number of anchoring fibrils, which secure the skin’s dermal layer to the epidermal layer [1]. Due to these mutations, patients with RDEB can experience blistering at the dermal layer with only minimal trauma.

An analysis of the United States (US) National Epidermolysis Bullosa (EB) Registry, funded and operated from 1986 to 2002, reported RDEB incidence of 3.05 cases per one million live births and prevalence of 1.35 cases per one million live births [2]. However, a more recent genotypic modeling of publicly available whole-exome and whole-genome sequencing estimated an incidence of 95 cases per one million births. This suggests that the National EB Registry estimates may be significantly understated, potentially due to underestimation of less severe cases of RDEB, likely mis-diagnosed as EB Simplex or de novo variants of Dominant Dystrophic EB (DDEB) [3]. This underestimation may also result in overestimation of systemic and severe manifestations.

RDEB is typically diagnosed clinically and often confirmed through assessment of immunofluorescence or electron microscopy on skin biopsy or by genetic testing [4]. RDEB is divided into several subtypes: severe (characterized by absent or markedly reduced C7), intermediate (characterized by reduced C7), and other, rarer, subtypes including inversa, localized, or pruriginosa [5].

Cutaneous signs of the disease include blistering and wounding in response to mechanical traumas, milia, atrophic scarring, dystrophic or absent nails, and alopecia. Pruritus is also a frequent complaint. Chronic wounding and fibrosis is generally believed to favor the frequent development of aggressive squamous cell carcinoma (SCC), which represents a leading cause of premature death in patients with severe and intermediate RDEB [4, 6].^1^ Extracutaneous manifestations include anemia, growth retardation, dental caries, pseudosyndactyly, esophageal strictures, malnutrition, and ocular involvement [4].

Currently, there are no approved disease-modifying therapies for RDEB. Treatment of the disease is limited to management of symptoms and secondary complications, such as wound care, prevention of trauma, treatment of infections, pain and itch management, strategic wrapping of the hands and feet to prevent pseudosyndactyly, and early detection and treatment of SCC [1, 7, 8]. Gastrointestinal manifestations of the disease are managed through proactive nutritional support including gastrostomy feeding, esophageal dilation, and treatment of anemia [1, 8]. Other areas of disease management include physical therapy and rehabilitation, psychosocial and group support, and extra schooling accommodations [1, 7, 9].

Due to the high unmet need for corrective treatments, the Food and Drug Administration released guidance for industry on the development of drugs for treatment of cutaneous manifestations of EB in June 2019, identifying drug development and trial design, population, and evaluation as issues specific to patients [10]. An increasing number of clinical trials are being conducted in cell-based therapies, gene and molecular therapies, protein replacement therapies, exon skipping molecular therapies, and drug-mediated premature termination codon read-throughs targeted to manage and treat RDEB [11]. Gene therapies, which involve the transfer of functional *COL7A1* gene to the patient with RDEB, appear to be promising potential treatments, likely available in the near future [12–14].

Many narrative and expert reviews provide a commentary on RDEB; however, no reviews use a systematic method to evaluate the literature regarding burden of disease in this patient population. This paper aims to systematically review and synthesize the data regarding the clinical, humanistic, and economic burden of RDEB. Abeona Therapeutics, a company developing EB-101, investigational autologous *COL7A1* gene-corrected keratinocytes sheets for the treatment of large and chronic RDEB wounds, initiated this review to fully characterize disease burden, and its employees (J.G and D.R.) co-authored the paper. Pharmerit conducted the literature review and generated the first draft.

## Methods

The systematic literature review was conducted in accordance with Preferred Reporting Items for Systematic Review and Meta-Analyses guidelines [15]. The literature review was developed based on a predefined search and selection protocol. Search terms are provided in Additional file 1: Table S1. The search focused on relevant studies published as journal articles or conference abstracts through April 2, 2020 in MEDLINE and Embase (searched via ProQuest), written in English.

Studies comprised of ≥ 80% patients with RDEB from any country were included. Observational studies (retrospective or prospective) including cohort, case–control, cross sectional studies, and case series and trials were included. Case reports (sample size < five patients), notes, editorials, and commentaries were excluded. Systematic reviews were included for the purpose of identification of primary studies. Animal and preclinical studies were excluded.

An experienced reviewer (EL, ST) independently screened all titles and abstracts resulting from the search methodology to identify articles for full-text review. Citations selected for full-text review were screened by the same reviewer for potential inclusion into the data extraction file and report. A second reviewer (EL, ST) verified the results of the title/abstract screening and full-text review. A standardized table was used to extract and record relevant data from selected publications.

## Results

### Summary of included studies

**Fig. 1:**
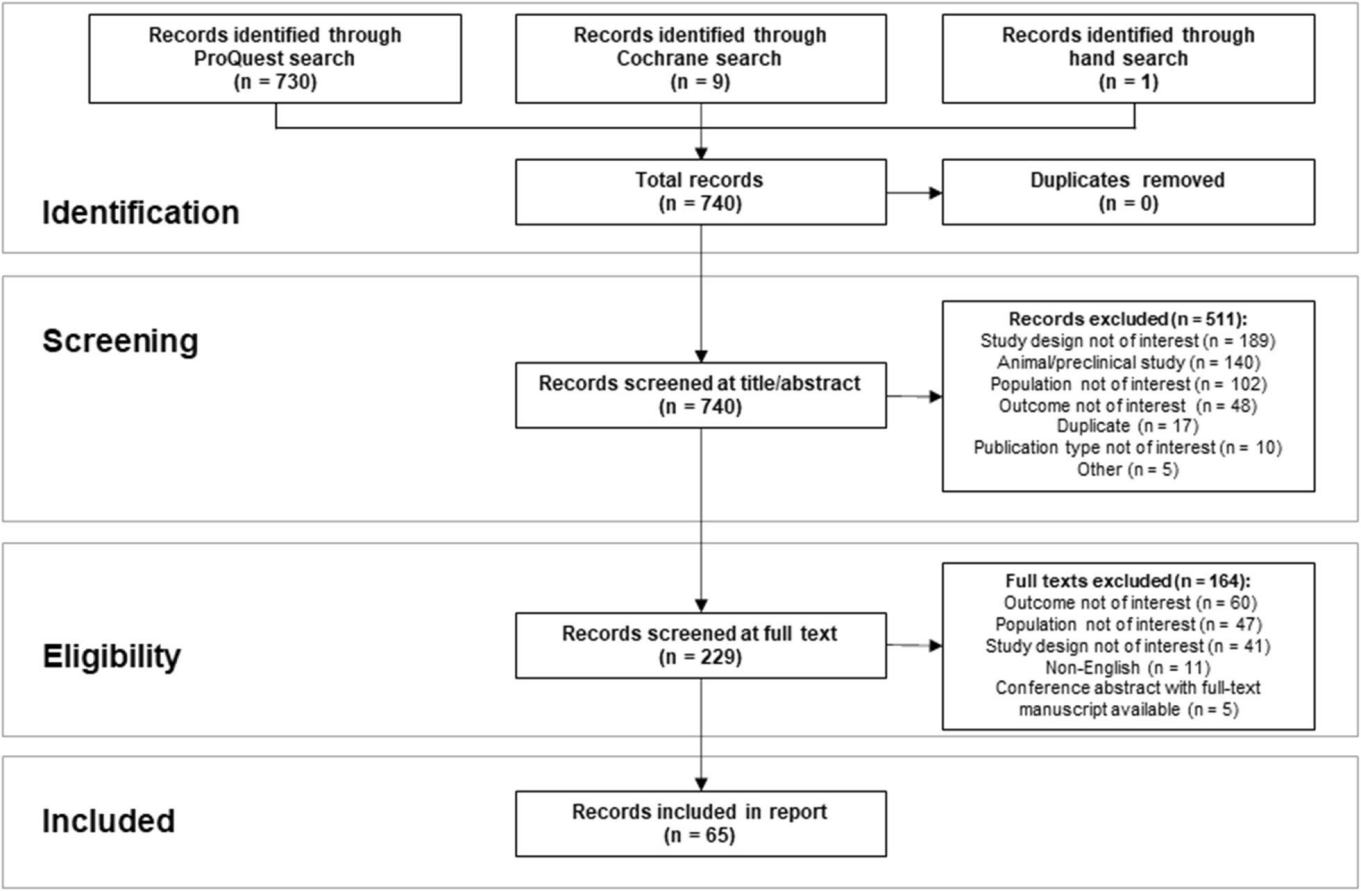
PRISMA study identification flow chart. PRISMA, Preferred reporting items for systematic review and meta-analyses

A total of 740 citations were identified, of which 229 full texts were screened and 65 were included within this review (56 manuscripts; nine conference abstracts or presentations) (Fig. 1; Additional file 1: Table S2). The majority of studies included all ages (n = 36 studies), or children (n = 20 studies); one study was in an adult-only cohort, and eight did not report age. The average age ranged between three years to 30 years of age at the time of the study. Most patients had severe or intermediate subtypes; the proportion of patients with severe RDEB included in the identified studies ranged from 26 to 100%.

### Clinical burden

#### Symptoms

Forty-one studies reported on symptom prevalence (Table 1) and/or burden [16–56]. Seven studies reported on the cumulative risk of symptoms at different ages including data from the US National EB Registry and Australasian EB Registry (Table 2) [6, 25–30].

**Table 1:** Incidence of symptoms in patients with RDEB

Symptom	%	n/N	Design, country
*Blisters/lesions*			
Blisters at or within 1 week of birth	94	15/16	Registry analysis, Australia/NZ [35]
	86	12/14	Registry analysis, UK [33]
	70	7/10	Single-center, Saudi Arabia [16]
*Oral lesions*			
Any oral lesions	100	35/35^a^	Multicenter, Spain [48]
	92	33/35^b^	Multicenter, Spain [48]
	89	8/9^c^	Registry analysis, UK [33]
	79	22/28	Single-center, Japan [43]
Lingual lesions	77	27/35	Multicenter, Spain [48]
Dental lesions	61	17/28	Single-center, Japan [43]
Soft palate lesions	60	21/35	Multicenter, Spain [48]
Oral bullae	59	10/17^d^	Case-review, Serbia [21]
Lesions on lips, mouth, tongue or ear	53	32/60	Single-center, Brazil [22]
Hard palate lesions	53	18/35	Multicenter, Spain [48]
Labial lesions	46	16/35	Multicenter, Spain [48]
Palatal milium cysts	46	16/35	Multicenter, Spain [48]
Jugal mucosa	34	12/35	Multicenter, Spain [48]
*Other lesions*			
Nail lesions	75	21/28	Single-center, Japan [43]
Lip lesions	53	32/60	Single-center, Brazil [22]
Esophageal lesions	47	28/60	Single-center, Brazil [22]
Nostril lesions	18	11/60	Single-center, Brazil [22]
Eyelid blisters	7	5/72	Single-center, UK [51]
External ear canal lesions	3	2/60	Single-center, Brazil [22]
Larynx lesions	2	1/60	Single-center, Brazil [22]
*Strictures/stenoses*			
Esophageal strictures/stenosis	86	6/7	Single-center, US [52]
	81	43/53	Survey, US [53]
	65	37/57	Single-center, UK [31]
	64	100/157	Single-center, Germany [55]
	64	53/83	Survey, International [54]
	51	216/424	Registry analysis, US [30]
*Other strictures/stenoses*			
Anal strictures	15	62/422	Registry analysis, US [30]
Pulmonary artery stenosis	14	1/7	Single-center, US [52]
Nostril stenoses	5	3/60	Single-center, Brazil [22]
Urethral meatal stenoses	3	14/425	Registry analysis, US [27]
Anterior commissure stenoses	2	1/60	Single-center, Brazil [22]
Pyloric stenoses or atresia	1	5/419	Registry analysis, US [30]
Laryngeal stenoses	0.7	3/412	Registry analysis, US [29]
Rectal strictures	0.2	1/422	Registry analysis, US [30]
*Malnutrition/failure to thrive*			
Malnutrition/nutritional problems	72	38/53	Survey, US [53]
	50	12/24	Single-center, France [20]
Failure to thrive	39	22/57	Single-center, UK [31]
	25	21/83	Survey, International [54]
Growth problems diagnosed by physician	34	18/53	Survey, US [53]
Negative height standard deviation scores	94	17/18	Single-center, UK [44]
Negative height velocity standard deviation scores	89	16/18	Single-center, UK [44]
*Nutritional deficiencies*			
Selenium deficiency	94	NR	Single-center, Germany [55]
Vitamin D deficiency	67	NR	Single-center, Germany [55]
Low albumin levels	56	NR	Single-center, Germany [55]
Zinc deficiency	55	NR	Single-center, Germany [55]
*Anemia*			
Any anemia	100	10/10	Single-center, Saudi Arabia [16]
	91	143/157	Single-center, Germany [55]
	76	40/53	Survey, US [53]
	68	17/25	Registry, Australia [34]
	60	47/79	Registry, UK [17]
	52	43/83	Survey, International [54]
	50	3/6	Single-center, US [52]
*Pseudosyndactyly*			
Any pseudosyndactyly	71	5/7	Single-center, Japan [43]
	50	14/28	Single-center, US [52]
	22	2/9	Single-center, Saudi Arabia [16]
Pseudosyndactyly of foot	55	46/83	Survey, International [54]
Pseudosyndactyly of hand	65	NR/425	Registry, US [28]
	13	11/83	Survey, International [54]
*Ocular symptoms*			
Any ocular symptoms	68	36/53	Survey, US [53]
	52	16/31	NR, Chile [42]
	52	43/83	Survey, International [54]
	51	37/72	Single-center, UK [51]
Corneal complications in those with ocular symptoms	100	16/16	NR, Chile [42]
	68	25/37	Single-center, UK [51]
	63	5/8^c^	Registry analysis, UK [33]
*Other ocular symptoms in those experiencing ocular involvement*			
Anterior blepharitis and collarettes	94	15/16	NR, Chile [42]
Corneal erosions	63	5/8^c^	Registry analysis, UK [33]
Symblepharon	59	8/16	NR, Chile [42]
Ectropion	38	6/16	NR, Chile [42]
	13	1/8^c^	Registry analysis, UK [33]
Conjunctival complications	14	5/37	Single-center, UK [51]
Exposure keratitis associated with upper and lower eyelid extropian’s	8	3/37	Single-center, UK [51]
*Other commonly reported symptoms*			
Nail dystrophy and loss	100	10/10	Single-center, Saudi Arabia [16]
	100	12/12	Registry analysis, UK [33]
Milia	100	9/9	Single-center, Saudi Arabia [16]
	93	49/53	Survey, US [53]
	21	6/28	Single-center, Japan [43]
Constipation	75	9/12	Registry, UK [33]
	72	38/53	Survey, US [53]
	60	254/422	Registry analysis, US [30]
	40	23/57	Single-center, UK [31]
Musculoskeletal contractures	87	46/53	Survey, US [53]
	67	4/6	Single-center, US [52]
	30	3/10	Single-center, Saudi Arabia [16]
Dental caries	24	54/225^e^	Case-review, Serbia [21]
*Infections*			
Any infection	64	53/83	Survey, International [54]
Skin infection	90	9/10	Single-center, Saudi Arabia [16]
Recurrent respiratory infection	50	5/10	Single-center, Saudi Arabia [16]
Bacterial septicemia	20	2/10	Single-center, Saudi Arabia [16]
Candida septicemia	10	1/10	Single-center, Saudi Arabia [16]

DEB, dystrophic epidermolysis bullosa; NR, not reported; NZ, New Zealand; RDEB, recessive dystrophic epidermolysis bullosa; UK, United Kingdom; US, United States

^a^Fibrotic lesion

^b^Blister lesion

^c^Population was patients with severe RDEB

^d^Population was children with DEB (88% RDEB)

^e^Of 225 permanent teeth in patients with RDEB

**Table 2:** Cumulative risk of symptoms over time in patients with RDEB

Symptom, study	Country, registry (date of data collection)	RDEB population (N)	Overall incidence (%)	Cumulative risk (%) at					
				1 year	10 years	15 years	20 years	40 years	60 years
*General symptoms*									
Esophageal stenoses and strictures	US, NEBR (1986–2002)	Severe (134)	79	7	57	72	79	89	95
Fine [30]		Intermediate (261)	37	4	27	34	40	62	70
		Inversa (15)	87	0	33	56	56	89	NR
Laryngeal stenoses and strictures	US, NEBR (1986–2002)	Severe (138)	2	0	1	1	1	5	5
Fine [29]		Intermediate (263)	0	0	0	0	0	0	0
		Inversa (17)	0	0	0	0	0	0	0
Pseudosyndactyly of the hands	US, NEBR (1986–2002)	Severe (142)	95	16	92	93	98	98	98
Fine [28]		Intermediate (266)	51	13	43	49	50	55	55
		Inversa (17)	41	0	8	26	26	26	26
Musculoskeletal contractures	US, NEBR (1986–2002)	Severe (142)	NR	13	83	92	99	NR	NR
Fine [28]		Intermediate (266)	NR	4	37	46	46	49	78
		Inversa (17)	NR	0	8	25	25	43	NR
CHF or cardiomyopathy	US, NEBR^a^ (1986–2002)	Severe (140)	7	1	2	4	7	19	19
Fine [25]		Intermediate (267)	1	0	0	0	1	3	3
Growth retardation	US, NEBR (1986–2002)	Severe (141)	NR	14	67	75	80	80	NR
Fine [30]		Intermediate (266)	NR	3	10	12	13	13	13
		Inversa (17)	NR	6	20	20	20	20	NR
*Premature mortality*									
Death from sepsis	US, NEBR^b^ (1986–2002)	Intermediate (262)	NR	0.4	0.4	0.4	NR	NR	NR
Fine [63]									
Death from pneumonia	US, NEBR (1986–2002)	Severe (138)	NR	0	0	1.8	NR	NR	NR
Fine [63]		Intermediate (262)	NR	0.4	0.4	1.1	NR	NR	NR
		Inversa (17)	0	0	0	0	NR	NR	NR
Death from respiratory failure	US, NEBR^a^ (1986–2002)	Severe (138)	NR	0	0	0.4	NR	NR	NR
Fine [63]		Intermediate (262)	NR	NR	NR	1.1	NR	NR	NR
Death from renal failure	US, NEBR (1986–2002)	All RDEB (417)	NR	0	0	0	NR	NR	NR
Fine [63]									
Death from failure to thrive	US, NEBR (1986–2002)	All RDEB (417)	NR	0	0	0	NR	NR	NR
Fine [63]									
Death from SCC	US, NEBR (1986–2002)	All RDEB (417)	NR	0	0	0	NR	NR	NR
Fine [63]									
*SCC-related*									
Development of SCC	UK, NEBR (2000–2015)	Children (79)	0	NR	NR	NR	NR	NR	NR
Alband [17]									
Development of SCC	US, Survey (2017)	Children (caregiver-reported) (34)	0	NR	NR	NR	NR	NR	NR
Bruckner [53]									
		Adults (19)	16	NR	NR	NR	NR	NR	NR
Development of SCC	Australia, AEBR^a^ (2009–2016)	Severe (11)	NR	NR	NR	NR	26	76^c^	NR
Kim [6]		Intermediate (5)	NR	NR	NR	NR	NR	10^c^	67^d^
Development of SCC	US, NEBR (1986–2002)	Severe (141)	23	0	0	0	8	74	NR
Fine [26]		Intermediate (263)	9	0	0	1	4	24	36
		Inversa (17)	18	0	0	0	0	8	NR
SCC-related death	Australia, AEBR^a^ (2009–2016)	Severe (11)	NR	NR	NR	NR	30^e^	84^f^	NR
Kim [6]		Intermediate (5)	NR	NR	NR	NR	NR	17^c^	67^ g^
SCC-related death (all patients with RDEB)	US, NEBR (1986–2002)	Severe (141)	NR	0	0	0	1	59	NR
		Intermediate (263)	NR	0	0	0	0	8	22
Fine [26]		Inversa (17)	NR	0	0	0	0	0	NR
SCC-related death (history of SCC)	US, NEBR (1986–2002)	Severe (32)	NR	0	0	0	13	81	NR
Fine [26]		Intermediate (24)	NR	0	0	4	4	31	60
		Inversa (3)	NR	0	0	0	0	0	NR

AEBR, Australasian Epidermolysis Bullosa Registry; CHF, congestive heart failure; NEBR, National Epidermolysis Bullosa Registry; NR, not reported; RDEB, recessive dystrophic epidermolysis bullosa; SCC, squamous cell carcinoma; US, United States

^a^Data not available in inversa subtype

^b^Data not available in severe or inversa subtype

^c^35 years

^d^65 years

^e^25 years

^f^34 years

^g^52 years

#### Wound burden

A US single-center study of 40 RDEB patients reported the clinical differences between recurrent or chronic open wounds [56]. Recurrent wounds healed but blistered again easily while chronic wounds remained open for 12 weeks or longer. Chronic wounds were significantly larger than recurrent wounds (66.3 cm^2^ vs. 44.7 cm^2^; *p* < 0.01) and more painful (4.31 of 10 points vs. 3.59; *p* = 0.05). Larger wound size was correlated with increased pain and itch among both chronic and recurrent wounds.

Results from a global registry survey of 85 RDEB patients with a total of 937 recurrent wounds and 289 chronic wounds reported a mean of 3 (SD, 2) chronic wounds and 11 (SD, 10) recurrent wounds per patient [54]. Recurrent wounds tended to be small (< 2.5 cm diameter; 491/937, 52%) or medium sized (2.5–7.5 cm; 355/937, 38%) rather than large (> 7.5; 91/937, 10%), while chronic wounds were generally evenly distributed between sizes (small: 88/289, 30%; medium: 103/289, 36%; large: 98/289, 34%). The majority of recurrent wounds took 0–1 weeks (197/937, 21%) or 1–3 weeks (702/937, 75%) to close while chronic wounds never closed (289/289, 100%). In a separate US survey, the majority of patients (N = 19) and caregivers (N = 34) reported that wounds covered more than 30% of the body (32/53, 60%); [53] 28% (15/53) reported wounds covering 10–30% of the body and only 11% (6/53) reported wounds covering less than 10% of the body.

In three studies of neonates with RDEB, the vast majority of infants developed blisters within one week of birth (range of 70% to 94%; Table 1) [16, 33, 35]. Five studies reported on the incidence of oral blisters or lesions in patients with RDEB [21, 22, 33, 43, 48]. The proportion of patients experiencing these lesions ranged from 79 to 100% [33, 43, 48]. Blisters and lesions of the nail, lip, esophagus, nostril, eyelid, ear canal, or larynx were reported in three studies [22, 43, 51].

#### Pain and itch

The burden of pain and itch was reported in seven studies [53, 54, 57–61], 5 of which utilized patient-reported outcome measures (PROMs, Table 5) [53, 57–60]. Patients with RDEB reported high levels of pain and pruritus compared to patients with DDEB, epidermolysis bullosa simplex (EBS), and other skin diseases [53, 57–60]. In a US survey study, patients with RDEB (N = 32) ranked the top three most bothersome symptoms to be skin lesions and blisters (7/32 [23%]), itching (5/32 [16%]), and pain (5/32 [16%]) [61]. A global survey of 83 patients with RDEB found that the majority of patients experienced itch (72/83, 85%), and presence of itch did not differ by patient-reported skin disease severity [54]. The worst pain experienced over the previous 12 months also did not differ by patient-reported skin disease severity in this cohort. Patients with RDEB assessing their pain via the Pain Quality Assessment Scale (PQAS) noted the highest scores (indicating increased pain/sensation) for unpleasant, sharp, intense, and tender pain [60].

#### Strictures and stenoses

In six studies, the proportion of patients with esophageal strictures ranged from 51 to 86% [30, 31, 52–55]. A Mexican analysis of 14 patients reported a median of one stenosis per year, with 74% (14/19 stenoses) in the proximal region [32]. A single-center UK study of 57 patients with stenoses reported a median of two stricture sites at esophageal dilation, the majority of which were located in the cervical or thoracic region (percentage not given) [41]. A single-center study in Croatia reported that in six patients, each with a stenosis, 83% (5/6 stenoses) were located in the upper third of the esophagus, with the remaining stenosis in the lower third [40].

Strictures and stenoses of the anus, pulmonary artery, nostril, urethra, anterior commissure, pylorus, larynx, and rectum were reported in 0.2% to 15% of patients with RDEB in five studies (Table 1) [22, 27, 29, 30, 52]. In two analyses of the US National EB Registry (N = 422–425), the lifetime cumulative risk of esophageal strictures was much higher than risk of laryngeal stenoses and strictures (Table 2) [29, 30]. Risk was higher in the severe subtype than the intermediate or inversa subtypes.

#### Malnutrition/failure to thrive

The proportion of patients with malnutrition/failure to thrive ranged from 25 to 72% while negative height and height velocity standard deviation scores were 94% and 89% (Table 1) [20, 31, 44, 53, 54]. In a UK analysis of 57 patients, etiologies of failure to thrive included reduction in dietary intake (due to dental involvement, pain from oral lesions), esophageal strictures, and heightened nutritional requirements secondary to extensive skin involvement [31]. A retrospective study of 157 German patients reported that approximately 50% of children with RDEB (exact numbers not reported) showed wasting (defined as weight below the third percentile) after the age of eight, and approximately 50% of children showed stunting (defined as height below the third percentile) after the age of ten; body mass index (BMI) in patients with RDEB fell in the underweight category (< 18.5 kg/m^2^), with a median BMI of 13.8 kg/m^2^ in men and 15.7 kg/m^2^ in women 20 years of age [55]. The nutritional characteristics of 12 patients with RDEB undergoing gastrostomy were described in a French, single-center study. Within the cohort, the mean estimated oral energy intake as a percentage of the recommended dietary allowance (adjusted for age and sex) was 56% (SD, 18) at time of gastrostomy feeding onset [20].

#### Anemia

The proportion of RDEB patients experiencing anemia ranged from 50 to 100% (Table 1), reported in seven studies [16, 17, 34, 52–55]. Mean hemoglobin levels ranged from 8.8. to 12.3 (Table 3) [34, 55, 62]. Analysis of 25 RDEB patients captured in the Australasian EB Registry found that 88% (22/25) required intermittent iron or blood transfusions to elevate hemoglobin levels [34].

**Table 3:** Anemia-related laboratory findings in patients with RDEB

ReferenceS	Population (N)	Mean (SD) hemoglobin (g/dL)	Mean (SD) reticulocytes (%)	Mean (SD) ferritin (µg/L)	Mean (SD) transferrin (mg/L)	Mean (SD) transferin saturation (%)	Mean (SD) iron (µg/L)
Reimer [55]	Children with RDEB (157)	9.7 (2.23)	17.8 (16.3)	63.0 (140.8)	241.7 (60.6)	9.9 (8.85)	27.6 (23.7)
	Normal range (NA)	12.55–16.55	4.8–16.4	22.5–275	200–360	16–45	26–151.5
Hwang [34]	Children with RDEB (NR)	10.19 (3.08)	NR	NR	NR	NR	NR
Mellerio [62]	RDEB severe, 0–16 years (NR)	10.84 (NR)	NR	NR	NR	NR	NR
	RDEB severe, 17–25 years (NR)	12.30 (NR)	NR	NR	NR	NR	NR
	RDEB severe, 26–35 years (NR)	11.09 (NR)	NR	NR	NR	NR	NR
	RDEB severe, 36–45 years (NR)	8.80 (NR)	NR	NR	NR	NR	NR
	RDEB severe, 46–55 years (NR)	9.30 (NR)	NR	NR	NR	NR	NR

NA, not applicable; NR, not reported; RDEB, recessive dystrophic epidermolysis bullosa

#### Pseudosyndactyly

The proportion of RDEB patients experiencing pseudosyndactyly ranged from 13 to 71% (Table 1), reported in three studies [16, 43, 52, 54]. The US National EB Registry reported the lifetime cumulative risk of pseudosyndactyly of the hands, which was highest in patients with the severe subtype (Table 2) [28].

#### Microstomia

The clinical burden of microstomia (abnormally small oral orifice) was reported in three studies [21, 48, 50]. A single-center case review of 17 Serbian patients up to age 21 years with DEB (88% RDEB) reported 77% of patients (N = 13) had microstomia, with an average mouth opening capacity of 40.1 mm (SD, 6.6) [21]. A Spanish multicenter case review found an average oral aperture in RDEB patients (N = 35) of 20.4 mm, compared to an average of 46 mm in healthy controls (N = 45) [48]. Eighty percent of RDEB patients had severe microstomia (oral aperture ≤ 30 mm) and 20% had moderate microstomia (31–40 mm). No patients with RDEB had mild microstomia (41–50 mm) or normal mouth opening size (≥ 40 mm). A Dutch single-center study reported 80% (8/10) of RDEB patients were unable to open their mouth wider than 35 mm [50]; the average maximal mouth opening in this patient cohort was 24.3 mm (SD, 11.6 mm).

#### Congestive heart failure and cardiomyopathy

An international multicenter case review (N = 13) reported a mean age of cardiomyopathy diagnosis of 12.6 years [39]. Notably, six (46%) of these patients were deceased at the time of publication. Reported in 407 patients with RDEB included in the US National EB Registry, the cumulative risk of dilated cardiomyopathy and congestive heart failure tended to be low over the patient lifetime, though risk in patients with severe subtype increased with age (Table 2) [25].

#### Ocular manifestations

The clinical burden of ocular symptoms in RDEB include corneal complications and erosions, anterior blepharitis and collarettes, symblepharon, ectropion, conjunctival complications, and exposure keratitis (Table 1) [33, 42, 51, 53]. The proportion of patients experiencing ocular involvement ranged from 51 to 68% [42, 51, 53, 54]. Of patients with ocular symptoms, the proportion with corneal complications ranged from 63 to 100% [33, 42, 51]. Other ocular symptoms were reported in three studies [33, 42, 51].

#### Other common symptoms and complications

Other commonly reported symptoms and complications, including nail dystrophy and loss, milia, dental caries, infections, constipation, and musculoskeletal contractures are reported in 12 studies (Table 1) [16, 17, 20, 21, 30, 31, 33, 34, 43, 52, 53, 55].

#### Premature mortality

In a UK registry analysis of 79 patients with RDEB aged 16 and younger, nine children (11%) died. Causes of death included sepsis and organ failure (n = 5), failure to thrive (n = 2), bowel perforation (n = 1), and preconditioning for bone marrow transplantation (N = 1) [17]. The US National EB Registry analyzed the cumulative risk of childhood death from pneumonia, sepsis, respiratory failure, renal failure, and failure to thrive as in Table 2 [63].

#### Squamous cell carcinoma

Database analyses of the cumulative risk of developing SCC showed low risk during childhood and increases with age, with a high risk for development of and mortality from SCC by 40 years (Table 2) [6, 17, 26, 53, 63]. In data from the US National EB Registry, SCCs tended to develop in chronic skin wounds (77.8% of SCC sites in 59 RDEB patients with SCC) [26]. The median number of SCC sites per patient was 3–3.5 (range: 1 to 40 sites). Median age at diagnosis, provided in a single-center study of 14 RDEB patients with SCC in Spain, was 24 years [64].

In an Australian registry analysis of patients with RDEB diagnosed with SCC (N = 16), the median number of SCC sites per patient was 7 and ranged from 1 to 56 sites, with a median age of 29.5 years at first SCC [6]. The majority of SCCs (95%; sample size not reported) developed on the extremities; 70% of those developed on the hands or feet. The site of SCC tended to be in areas of chronic ulcers and non-healing wounds, though percentages were not provided. Almost 70% (11/16) of patients diagnosed with SCC experienced metastasis to regional lymph nodes (100% of patients with metastasis, 10/10 patients [data not available in one patient]), lungs (80%, 8/10), vertebrae (30%, 3/10), and liver, adrenal gland, and muscle (10%, 1/10) over the patient lifetime. Almost half of patients included in the study (7/16, 44%) underwent therapeutic amputation in their lifetime; median age at first amputation was 29 years.

In a single-center study in Spain, 35% of patients (8/23) underwent amputation due to SCC [64]. In a study of 59 patients with RDEB and SCC, surgical amputation of at least one limb was performed in 21% of patients with the intermediate subtype to 42% with the severe subtype of patients (sample size of subtypes not reported) [26]. Amputation of the leg was most common (intermediate subtype, 67%; severe subtype, 29%), followed by arm (intermediate subtype, 33%; severe subtype, 29%), hand (intermediate subtype, NR; severe subtype, 29%), and foot (intermediate subtype, NR; severe subtype, 14%).

#### Procedures

Esophageal dilation and gastrostomy tube placement were the procedures most frequently reported upon in the literature. Ten studies reported on the use of esophageal dilations in patients with RDEB [17, 20, 23, 30, 40–42, 55, 65, 66], and ten studies reported on the use of gastrostomy tubes (Table 4) [17, 23, 30, 31, 39, 49, 54, 55, 65, 66]. One single-center case review in the UK reported on treatment burden and satisfaction associated with gastrostomy tubes in RDEB (N = 57) [65]. Two-thirds of children with RDEB (10/15) and all of their parents (15/15) reported a satisfaction level with the gastrostomy tube of at least seven out of ten (indicating extreme satisfaction) over a median 8.9 years since placement. Almost half of patients (7/15, 47%) reported no gastrostomy site infections in the previous year; one-third reported fewer than two infections in the previous year (5/15, 33%). The remaining patients reported either 2–4 infections (n = 2) or constant infections in the previous year (n = 1).

**Table 4:** Procedures in Patients with RDEB

Variable	Data	Design, Country
*Esophageal dilation (ED)*		
Proportion undergoing ED, % (n/N)	74 (23/31)	NR, Chile [42]
	56 (157/283)	Registry analysis, US [23]
	43 (34/79)	Registry analysis, UK [17]
	38 (NR/25^e^)	Single-center, US [66]
	33 (134/411)	Registry analysis, US [30]
	29 (45/157)	Single-center, Germany [55]
Average EDs performed per patient, mean/median (N)	Mean, 7 [14]	Single-center, UK [65]
	Median, 6 (77)	Single-center, UK [41]
	Median, 5 (17, inversa subtype)	Registry analysis, US [30]
	Median, 3 (136, severe subtype)	
	Median, 2 (258, intermediate subtype)	
Maximum number of EDs performed per patient, no (N)	14 (14)	Single-center, UK [65]
	41 (77)	Single-center, UK [41]
	50 (411)	Registry analysis, US [30]
Age at first ED, years (N)	5.5 (77)	Single-center, UK [41]
*Gastrostomy tube*		
Proportion undergoing GT, % (n/N)	58 (33/57)	Single-center, UK [31]
	37 (104/283)	Registry analysis, US [23]
	33 (27/83)	Survey, International [54]
	32 (25/79)	Registry analysis, UK [17]
	24 (97/412)	Registry analysis, US [30]
	14 (22/157)	Single-center, Germany [55]
	8 (2/25^e^)	Single-center, US [66]
Average GTs performed per patient, median (N)	Median, 1 (412)	Registry analysis, US [30]
Maximum GTs performed per patient, no (N)	10 (412)	Registry analysis, US [30]
Age at first GT, years (N)	6 (6^a^)	Single-center, UK^24^
	8 (44^a^)	Single-center UK^36`^

ED, esophageal dilation; GT, gastrostomy tube; No, number; NR, not reported; RDEB, recessive dystrophic epidermolysis bullosa; UK, United Kingdom; US, United States

^a^Children with RDEB

#### *Pseudosyndactyly* release

Three studies reported on pseudosyndactyly release of the hands or feet in patients with RDEB [23, 28, 66]. Within a US and Canadian cohort of 238 RDEB patients, 62 (22%) underwent hand surgery. The median age for their first hand surgery was 8.1 years (IQR, 5.5–12.1 years; range, 3–25 years) [23]. Among 414 patients in the US National EB Registry, 151 (37%) underwent mitten repair of the hands and 11 (3%) underwent mitten repair of the feet [28]. The median number of hand surgeries performed was 3.0 (range: 1–22 surgeries). Data on the median number of foot surgeries was not available. A retrospective analysis of 25 children with RDEB reported that 27% of patients (sample size not provided) underwent pseudosyndactyly release with or without skin graft [66].

#### Diagnostic procedures

Analysis of 283 RDEB patients in the EB Clinical Characterization and Outcomes Database reported that confirmatory diagnostic testing was performed in 77% (218/283) of patients, and 63% (178/283) underwent multiple methods of diagnostic testing [23]. Of all RDEB patients, 65% (184/283) underwent genetic analysis, 41% (116/283) immunofluorescence, and 35% (98/283) electron microscopy.

### Humanistic burden

#### Patient-reported outcome measures (PROMs)

Eighteen studies utilized 16 distinct PROMs (Table 5) [19, 24, 47, 53, 54, 57–61, 67–74]. The most commonly used PROMs were the Quality of Life in Epidermolysis Bullosa survey (QOLEB) [54, 59, 61, 69–71], the Visual Analogue Scale (VAS) for pain or pruritus [58, 59], and the instrument for scoring clinical outcomes of research for epidermolysis bullosa (iscorEB) [68, 72].

**Table 5:** Patient-reported outcome measures utilized in patients with RDEB

PROM	Brief description	Study, country	Patients w RDEB (n)	Results
*Dermatology-specific instruments*				
QOLEB	First disease-specific QOL tool for EB 17 items with scores ranging from 0 (least impact) to 3 (most impact) Overall QOL scores range from 0 to 51 Lower values indicate better function/higher QOL	Cestari [69], Brazil	13 (child) 6 (adult)	Children with RDEB reported lowest overall QOL (14.3 [SD, 9.7]) followed by EBS (10.6 [7.1]), DDEB (9.7 [7.9]), and JEB (5.0 [NA]); non-significant difference Adults with RDEB (20.2 [9.2]) reported lowest overall QOL followed by EBS (12.5 [10.0]), DDEB (12.0 [5.8]), and JEB (2.0 [NA]); non-significant difference
		Choi [61], US	32	Patients reported frequent or constant pain (69%) and a high or severe level of psychological and social impact on finances (50%), friendships (41%), anxiety (41%), depression (31%), family (22%), and embarrassment (16%) Patients reported severely impaired function in eating (63%), bathing (53%), moving outside the home (38%), writing (28%), and moving around the home (22%)
		Eismann [70], US	32	Children with RDEB reported lowest overall QOL (23 [IQR, 13–27]) followed by JEB (21 [13–26]), EBS (19 [5–30]), and DDEB (13 [6–18]); significance not measured Items associated with the worst QOL (score ≥ 2) include bathing/showing (2.45 [SD, 2.45]), sports (2.45 [0.68]), physical pain (2.06 [0.95]), and eating (2.06 [0.95])
		Eng [54], International	81	QOLEB score in patients with RDEB did not vary significantly by patient-reported disease severity (mild [mean, 19, SD, 3.4], moderate [7, 20], severe [6, 24]); * p* = 0.36 QOLEB score significantly differed by the size of patients’ predominant wounds; patients with large wounds (> 7.5 cm) had worse QOL (median score, 27) than patients with medium wounds (2.5–7.5 cm; median score, 22.5) or small wounds (< 2.5 cm; median score, 14); * p* = 0.02
		Frew [71], Australia	16	Patients with RDEB reported worst overall QOL (35.5 [SD, 12.7]) followed by JEB (31.5 [17.6]), DDEB (18.1 [10.9]), and EBS (13.7 [8.7]); significance not reported
		Jeon [59], South Korea	13	Patients with severe subtype of RDEB (N = 7) reported worse overall QOL (30.1 [SD, 8.8]) than those with intermediate (N = 6; 23.2 [3.8]); non-significant difference Patients with very severe perceived disease (N = 11) reported lower overall QOL (28 [7.8]) than those with severe perceived disease (N = 2; 21 [NA]); * p* < 0.05 Patients with RDEB hospitalized for > 7 days (N = 5) in the past year reported lowest overall QOL (29.8 [8.7]) compared to patients hospitalized 1–6 days (N = 1; 25 [NA]) or 0 days (N = 7; 25.14 [7.35]); non-significant difference
iscorEB	Comprised of clinical score (5 domains) and patient score (7 domains) Scores range from 0–120 Designed to capture changes over time Higher score indicates worse burden	Bruckner [68], US	16	Mean clinical and patient scores significantly higher in patients with RDEB (clinical, 19.9; patient, 41.0) than other subtypes of EB (clinical, 3.2; patient, 24.7) * p* < 0.0001 (clinical difference) and * p* = 0.004 (patient difference)
		Schwieger-Briel [72], Canada	NR	Patients with severe subtypes of EB significantly lower QOL (64.5 [SD, 22.6]) than those with moderate (41.0 [19.4]) or mild subtypes (17.3 [9.6]); * p* < 0.001 Patients with DEB report significantly lower QOL (57.2 [24.6]) than patients with EB (30.6 [19.2]); * p* = 0.007
Birmingham EB Severity score	Method of scoring clinical severity Scores range from 0–100 Higher score indicates worse burden	Moss [74], UK	34	Patients with the severe subtype of RDEB had higher median score (22.9 [range, 2.8–27.8]) than those with non-severe subtype (7.8 [2.8–27.8]) Scores in the severe subtype of RDEB were significantly associated with age (demonstrating disease progression); * p* = 0.001
FDLQI	Measures adverse impact of HRQOL on family members with disease 10 questions on 4-point scale Scores range from 0–30, higher score indicates worse QOL Not specific to EB	Sampogna [47], Italy	62	QOL was significantly worse in patients with severe disease (14.3) or moderate disease (11.4) than mild disease (3.4); * p* < 0.001 QOL was significantly worse in patients with > 30% of their body involved (14.4) and 10–30% involved (10.9) than < 10% involved (6.3); * p* = 0.003 QOL was significantly worse in caregivers who were mothers (10.6) than non-mother caregivers (5.4); * p* = 0.025 QOL was significantly worse in patients with probable anxiety or depression (measured via GHQ-12; 13.9) than in those without (8.2); * p* = 0.003 QOL was similar between male (9.4) and female sex (10.1); non-significant difference QOL was slightly worse in patients with a disease duration of 10 years or more (10.9) compared to a duration of less than 10 years (7.7); non-significant difference Most frequently reported problems include time spent looking after the patient, emotional distress, affected physical well-being, and increased household expenditure (exact frequencies not reported)
InToDermQOL	Parent-reported measure for children with skin diseases aged 0–4 years Undergoing item testing and validation Not specific to EB	Chernyshov [19], Ukraine and Romania	12	Over half of parents of infants and toddlers with RDEB mentioned itching (12/12 [100%]), problems with defecation (11/12 [92%]), problems with feeding (9/12 [75%]), pain (8/12 [67%]), sleep problems (7/12 [58%]), and treatment (7/12 [58%]),
Skindex-29	29 items comprising 3 scales (symptom, functioning, emotional burden) Scores range from 0–100 Higher scores indicate worse QOL Not specific to EB	Jeon (59), South Korea	13	Patients with RDEB had the highest symptom scale score (86 [SD, 10]; Fig. 2b) compared to patients with vulvodynia (50 [17]), eczema (48 [23]), dermatomyositis (42 [25]), psoriasis (42 [21]), rosacea (33 [20]), alopecia (31 [24]), and acne vulgaris (30 [19]) as well as people without skin disease (14 [2]); significance not reported Patients with RDEB had the highest emotion scale score (75 [16]; Fig. 2b) compared to patients with vulvodynia (50 [20]), dermatomyositis (45 [27]), eczema (41 [27]), acne vulgaris (41 [25]), psoriasis (39 [27]), rosacea (33 [20]), and alopecia (27 [33]) as well as people without skin disease (9 [13]); significance not reported Patients with RDEB had the highest function scale score (77 [12]; Fig. 2b) compared to patients with vulvodynia (44 [22]), dermatomyositis (28 [29]), eczema (26 [26]), psoriasis (23 [27]), acne vulgaris (16 [16]), rosacea (16 [18]), and alopecia (14 [23]) as well as people without skin disease (4 [8]); significance not reported
*Generic instruments*				
Instruments measuring physical functioning				
ABILIHAND	Individual item scores range from 0 (impossible) to 2 (easy) Overall hand function score ranges from 0 to 42 Higher score indicates better hand function	Eismann [70], US	32	Difficult to impossible items (score ≤ 1) for children with RDEB included opening a jar of jam (0.17 [SD, 0.38]), buttoning up pants (0.43 [0.57]), opening a bag of chips (0.43 [0.63]), buttoning up a shirt or sweater (0.45 [0.57]), unscrewing a bottle cap (0.50 [0.72]), fastening the snap of a jacket (0.77 [0.63]), zipping up pants (0.90 [0.76]), switching on a bedside lamp (0.93 [0.74]), zipping up a jacket (0.97 [0.65]), sharpening a pencil (0.97 [0.78], rolling up a sleeve of a sweater (0.97 [0.82]) Children with RDEB reported lowest hand function (21 [IQR, 17–29]), followed by EBS (28 [21–36]; * p* = 0.031), JEB (30 [22–37]; * p* = 0.014), then DDEB (40 [36–42]; * p* < 0.001)
ADLs	First application in skin diseases 109 items Rates levels of independence in performing activities of daily living	Fine [58], US	45	Children with RDEB reported being totally dependent at bathing (27%), grooming (20%), dressing (13%), and walking (13%) Children with RDEB reported being totally independent at feeding (73%), toileting (71%), bathing (47%), dressing (42%), grooming (42%), and walking (24%) Children with RDEB reported high levels of dependence in bathing (27% totally dependent), grooming (20%), dressing (13%), and walking (13%), similar to children with DEB (bathing, 27%; grooming, 19%; dressing, 15%; walking, 8%); significance not reported Children with DEB and EBS reported low levels of dependence (DDEB, 0% throughout; EBS, 2% totally dependent in bathing, grooming and walking)
Instruments measuring performance and mental health				
Achenbach’s Child Behavior Checklist	Parent-reported measure T-scores for respective sex/age group available (50 being normal)	Feldmann [24], Germany	9^a^	Parents of children with RDEB severe subtype and children with other subtypes of EBS (including RDEB intermediate) reported no significant differences between groups in total competence score (38.3 [SD, 14.3] vs. 43.3 [7.8]), internalizing (62.4 [8.9] vs. 57.1 [15.1]), externalizing (49.9 [7.7] vs. 56.9 [10.3]), and total problem score (59.3 [8.8] vs. 58.8 [12.5]); non-significant difference All scores were in the normal range
GHQ-12	12-items Designed to detect presence of minor non-psychotic psychiatric disorders	Sampogna [47], Italy	62	QOL was significantly worse in patients with probable anxiety or depression (measured via GHQ-12; 13.9) than in those without (8.2); 0.003
Graphic tests	Projective test designed to measure personality	Andreoli [67], Italy	11	When measuring intellectual development, all children with RDEB were labeled normal (18%) or above normal (82%) intellectual development. All children with EBS, JEB and DDEB were labeled as above normal; significance not reported When measuring affective development, a higher proportion of children with RDEB were labeled with immaturity (46%) than maturity (36%) or forced growth (18%). All patients with EBS and DEDB and 80% of patients with JEB were labeled as mature; significance not reported When measuring scholastic/working efficiency, a higher proportion of children with RDEB were labeled as adequate or high (73%) than inadequate (27%). All patients with EBS, JEB and DDEB were labeled as adequate or high; significance not reported When measuring drive display, a higher proportion of children with RDEB were labeled as adequate (64%) than coarctate (18%) or excessive (18%). All patients with DDEB, 80% of patients with JEB, and 33% of patients with EBS were labeled as adequate; significance not reported When measuring psychosocial development, a higher proportion of children with RDED were labeled with accommodating adjustment (46%) or assimilative adjustment (36%) than reported maladjustment (18%). All patients with EBS and DDEB and 80% of patients with JEB were labeled as accommodating or assimilative adjustment; significance not reported
Strengths and difficulties questionnaire	Completed by caregiver Includes 6 subscales and combined total difficulties scale	Soon [73], UK	18	Parents reported higher proportion of children with RDEB than children with EBS scored in clinical range for emotional symptoms (66% vs. 50%) and peer-relationship problems (50% vs. 40%); significance not reported Children with RDEB 2–3 × more likely to have clinically significant difficulties in these areas than a non-RDEB population
Wechsler Intelligence Scale	Separate scales for children and adults	Feldmann [24], Germany	9^a^	Children with the RDEB severe subtype reported significantly lower performance (75.6 [SD, 18.1] vs. 99.2 [14.7]), verbal (77.6 [16.7] vs. 101.6 [9.4]) and full scale scores (74.3 [18.0] vs. 100.6 [12.5]) than patients with other subtypes of EB (including RDEB intermediate subtype); * p* < 0.05
Instruments measuring pain and itch				
Pain and pruritus scales^b^	Measured on a scale of 1 to 10 with higher scores indicating higher/more frequent pain or itch	Bruckner [53], US	19 (patient) 34 (caregiver)	Patients with RDEB reported higher acute pain (5.6 [SD NR]) than patients with DDEB (4.4), JEB (4.4), or EBS subtypes (4.3); significance not reported Patients with RDEB reported similar chronic pain (5.3) to patients with JEB (5.3) and higher chronic pain than patients with DDEB (4.6) or EBS (3.2); significance not reported Patients with RDEB reported similar levels of itch (6.7) to patients with JEB (6.5) and higher levels of itch than patients with DDEB (5.5) or EBS (4.4); significance not reported Caregivers reported their patients with RDEB had similar levels of acute pain (6.4) to patients with JEB (6.3) and higher levels of acute pain than patients with EBS (5.3) or DEB (5.0); significance not reported Caregivers reported their patients with RDEB had similar levels of chronic pain (4.2) to patients with JEB (4.0) and higher levels of chronic pain than patients with EBS (3.6) or DDEB (3.4); significance not reported Caregivers reported their patients with RDEB had higher levels of itch (7.2) than patients with EBS (4.4), DDEB, (4.3), and JEB (4.0); significance not reported
	Measured on a 5-point Likert scale with higher scores indicating more frequent itch	Danial [57]	77	When asked frequency of itch per day, patients with RDEB reported the highest scores (3.9 [SD, 0.8]) compared to patients with JEB (3.6 [0.8]), DDEB (3.5 [1.2]), and significantly higher than EBS (3.1 [1.0]); * p* = 0.01 Patients with RDEB reported highest itch at bedtime (4.0 [NR]) Itch was the most bothersome symptom of EB (3.3 [1.1]), compared to acute pain (2.9 [1.3]), chronic pain (2.7 [1.5]), and problems eating (2.7 [1.4]) Itch was more bothersome in RDEB patients (3.5) than DDEB (3.1) and EBS (2.7) Itch was most severe in patients with self-reported severe disease (4.0 [0.8]), compared with moderate EB (3.8 [0.9]), and mild EB (3.2 [1.0])
PQAS	Scores range from 0 (no pain/sensation) to 10 (most pain/sensation)	Schräder [60], Netherlands	5	Patients with RDEB (compared to EBS) reported significantly higher levels of unpleasant (6.4 [3.5] vs. 3.5 [2.6]), intense (5.4 [2.8] vs. 2.4 [2.4]), surface (5.2 [2.6] vs. 2.4 [2.3]), itchy (4.8 [3.3] vs. 2.1 [2.5]), sharp (5.8 [2.9] vs. 1.7 [2.1]) and shooting pain (5.2 [2.5] vs. 1.3 [2.2]); * p* < 0.05 Patients with RDEB reported higher unpleasant (6.4 [3.5] vs. 4.5 [3.0]), sharp (5.8 [2.9] vs. 3.4 [3.3]), intense (5.4 [2.8] vs. 3.8 [3.1]), tender (5.4 [3.6] vs. 3.7 [2.9]), surface (5.2 [2.6] vs. 3.7 [2.9]), deep (5.2 [4.0] vs. 3.4 [3.1]), shooting (5.2 [2.5] vs. 2.7 [3.2]), itchy (4.8 [3.3] vs. 3.5 [3.5]), heavy (4.8 [3.4] vs. 3.3 [3.7]), hot (4.6 [3.9] vs. 2.8 [3.0]), aching (4.4 [3.2] vs. 3.4 [3.2]), sensitive (4.0 [2.6] vs. 3.4 [2.6]), dull (4.0 [3.9] vs. 2.5 [2.9]), tingling (4.0 [3.4] vs. 2.5 [2.8]), throbbing (3.8 [3.6] vs. 3.0 [2.9]), radiating (3.8 [3.8] vs. 2.1 [2.87]), cramping (3.6 [3.5] vs. 1.7 [2.3]), and cold pain (2.0 [2.3] vs. 1.0 [1.9]) than all EB types; significance not reported
VAS	Linear, visual analog scale from 0 (no pain/itch) to 10 (most severe pain/itch) Differences of 6–10 mm considered clinically meaningful	Fine [58], US	45 (child) 35 (adult)	A higher proportion of adults with RDEB reported an average pain severity of greater than 5 points (26%) than adults with EBS (18%), DDEB (8%), but a lower proportion than adults with JEB (33%); significance not reported A higher proportion of children with RDEB reported an average pain severity of greater than 5 points (32%) than children with EBS (19%), DDEB (14%), or JEB (15%); significance not reported
		Jeon [59], South Korea	13	Patients with RDEB reported a higher mean score on the VAS-pain (6.54 [SD, 1.56]) than patients with herpes zoster (5.20 [1.61]) or oral lichen planus (4.12 [0.36]); significance not reported Patients with RDEB reported a similar but slightly lower mean score on the VAS-pruritus (7.54 [2.07]) than patients with prurigo nodularis (8.0 [1.7]), chronic urticaria (7.9 [1.44]), and atopic dermatitis (7.9 [2.2])

ADL, activities of daily living; DDEB, dominant dystrophic epidermolysis bullosa; EB, epidermolysis bullosa; EBS, epidermolysis bullosa simplex; FDLQI, Family Dermatology Life Quality Index; InToDermQOL, Infants and Toddlers Dermatology Quality of Life; GHQ-12, General Health Questionnaire-12; HRQOL, health-related quality of life; iscorEB, instrument for scoring clinical outcomes of research for epidermolysis bullosa; JEB, junctional epidermolysis bullosa; PQAS, Pain Quality Assessment Scores; QOL, quality of life; QOLEB, Quality of Life in Epidermolysis Bullosa; RDEB, recessive dystrophic epidermolysis bullosa; UK, United Kingdom; US, United States; VAS, visual analog scale; SD, standard deviation

^a^Children with severe subtype of RDEB

^b^Specific measurement names not reported

Some studies [24, 47, 58, 59, 70] report on multiple PROMs and are listed multiple times within the table

**Fig. 2:**
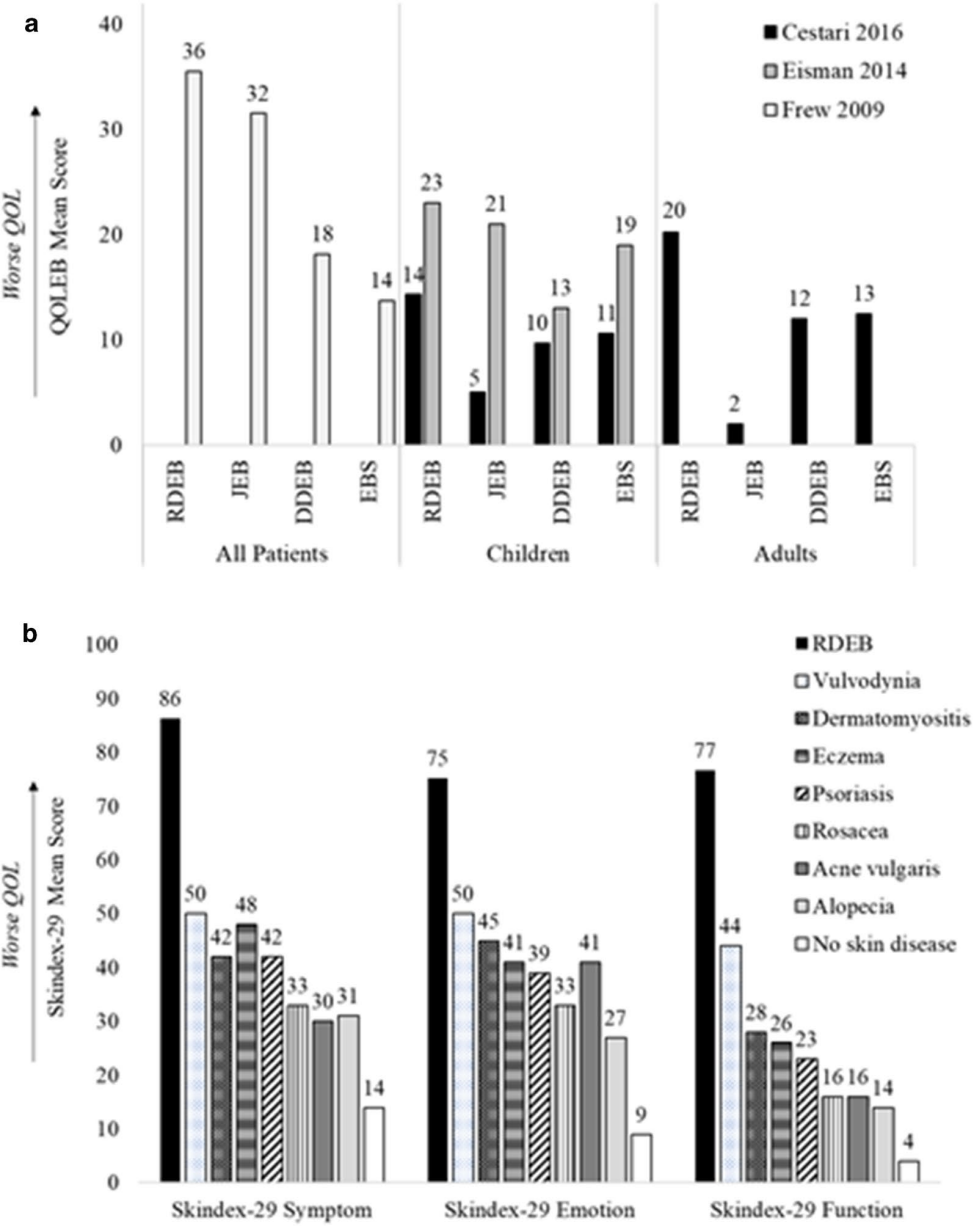
Differences in QOL between **a** EB subtypes (via QOLEB^a^), **b** skin diseases (via Skindex-29b). Adapted from: Cestari [69], Eisman [70], Frew [71], Jeon [59]. DDEB, dominant dystrophic epidermolysis bullosa; EB, epidermolysis bullosa; EBS, epidermolysis bullosa simplex; JEB, junctional epidermolysis bullosa; QOL, quality of life; QOLEB, Quality of Life in Epidermolysis Bullosa; RDEB, recessive dystrophic epidermolysis bullosa. aQOLEB is an EB-specific patient-reported outcome measure with scores ranging from 0 (best possible function/highest possible QOL) to 51 (lowest possible function/worst possible QOL). bSkindex-29 is a dermatology-specific patient-reported outcome measure with scores ranging from 0 (best possible QOL) to 100 (worst possible QOL)

Patients with RDEB experienced significant impairment in overall quality of life (QOL) across multiple PROMs and domains (Table 5). RDEB patients had lower QOL than patients with other EB subtypes and patients with other skin diseases, especially compared with patients with more common diseases such as atopic dermatitis and psoriasis (Fig. 2).

#### Functioning and social activities

Patients with RDEB experienced limitations in functioning and social activities; many patients with RDEB required assistance or are unable to complete activities of daily living (Table 5). A US survey of RDEB patients (N = 19) and caregivers of RDEB patients (N = 34) reported an impact on the patient’s ability to play (50/53, 94%), sleep (47/53, 89%), eat (45/53, 85%), move around the home (44/53, 83%), bathe or shower (42/53, 79%), shop (33/53, 62%), and write (28/53, 53%) due to their disease [53].

#### Impact on families and caregivers

The humanistic burden of disease extended beyond patients to affect their families. In a US study of parents of children with RDEB (sample size not provided), 90% reported that their ability to remain physically and emotionally close to their significant other was negatively impacted by their child’s condition [75]. Additionally, over three-quarters (79%) reported that their private life had suffered and 64% chose not to have more children due to their child’s illness. Fifty-nine percent reported that their relationship was negatively affected by their child’s illness, and 50% had little energy to do more than care for their child. Of parents who divorced (22%), 67% reported that their child’s disease was a major, if not primary, influencing factor in their divorce, and 30% cited the financial burden of their child with RDEB as the reason for divorce. In an Italian registry analysis of 62 patients with RDEB and their family caregivers (sample size not reported), the most frequently reported problems among caregivers were the time spent looking after their children with RDEB, emotional distress, worsened physical well-being, and increased household expenditure [47].

### Economic burden

Eight publications reported on economic outcomes in patients with RDEB or their families [53, 59, 61, 62, 76–79]. Costs of wound care (including dressing and time costs), medical costs, and hospitalization costs were reported in South Korea [59], Ireland [78], the United Kingdom (UK) [62, 76], and the US [77].

#### Direct costs and healthcare resource use

Direct medical costs in patients with RDEB were high (Table 6) [59, 62, 76–78]. Medical expenses varied considerably; a patient survey in Ireland (N = 5) reported median payer-borne total medical costs, consisting of costs for wound dressings, drugs, overnight hospital stays, and outpatient visits, to be $84,534 per year [78], and a patient survey in South Korea (N = 13) reported total patient-borne medical costs, comprising medical dressings and all other disease-related expenses, to be $7392 per year [59]. Costs of wound dressing materials ranged from $4000-$245,000 in a US cost exercise model incorporating patient age and material quality [77], while costs of dressing materials reported in patient surveys from Ireland, South Korea, and the UK ranged from $4296-$28,727 [59, 62, 76]. Variations in cost are likely due to small sample sizes, contrasting health systems, and differences in EB subtypes. Patients with the severe subtype or complex wounds tended to report higher expenses [62, 76, 78].

**Table 6:** Dressing- and medical-related expenses per patient per year

Citation, study design	Country	Patient population	Sample size, N	Cost per patient per year^a^	Cost year	Definition
*Dressing costs*						
Jeon [59]	South Korea	RDEB	13	$4296	2016 USD^c^	Dressings, fixing materials, topical agents and medicines used during changes
Patient survey^b^						
Mellerio [62]	United Kingdom	RDEB	40	$9049	2016 GBP^c^	Cost of dressing
Patient and caregiver survey^b^		RDEB, severe	17	$17,151		Cost of dressings
		RDEB	11	$15,293		Cost of hours spent dressing wounds
Grocott [76], single-center, cross-sectional survey^b^	United Kingdom	RDEB, with wounds difficult to manage with conventional dressings	11	$28,727	2012 GBP	Dressing materials, costs estimated via monthly dressing orders
Kirkorkian [77],	United States	RDEB, neonate	NA	$4,000–$47,000	2014 USD	Cost of wound care products obtained from Amazon.com (August 2012 prices) based on body-size
Cost exercise model		RDEB, infant		$8,000–$99,000		
		RDEB, 10 year old		$20,000–$245,000		
Flannery [78], Patient survey^b^	Ireland	EB	5 (4 RDEB)	$32,256	2020 EUR^c^	Median wound and drugs cost
*Medical, non-dressing-related costs*						
Jeon [59], Patient survey^b^	South Korea	RDEB	13	$3096	2016 USD	All RDEB expenses excluding dressing costs
Mellerio [62], Patient and caregiver survey^b^	United Kingdom	RDEB	10	$1249^d^	2016 GBP^c^	Cost per hospital stay, assuming ₤212 per day
Flannery [78], Patient survey^b^	Ireland	EB	5 (4 RDEB)	$84,534	2020 EUR^c^	Median total medical costs
				$33,679		Median overnight hospital costs, assuming €813 per night
				$2890		Median day clinic costs, assuming €407 per visit
				$1304		Median other primary care costs, including GP visits, physiotherapy, occupational therapy, public health nurse visits

GBP, British pound sterling; GP, general practitioner; EB, epidermolysis bullosa; EUR, euro; NA, not applicable; NR, not reported; RDEB, recessive dystrophic epidermolysis bullosa; USD, United States dollar

^a^All costs converted to USD based on November 5, 2020 exchange rate

^b^Patient surveys were used to gather healthcare resource utilization and then local unit costs were applied to generate cost estimates

^c^Year of currency not defined, assumed to be publication year

^d^Cost reported per hospital stay not per year

Hospital resource use was reported in two studies in South Korea [59] and the UK [62], both with small samples. Almost half of patients in a South Korean survey were hospitalized in the previous year due to RDEB (6/13, 46%) [59]. Five patients (39%) were hospitalized for more than seven days. A survey of UK patients with RDEB (N = 10) reported a median duration of hospital stay of four and a half days (range: 2–155 days) [62].

#### Non-direct medical costs

##### Frequency of dressing changes

RDEB patients required frequent dressing changes [53, 59]. Seven of the 13 (54%) patients included in the Korean survey reported daily dressing changes; two (31%) reported dressing changes three times per week [59]. In a US survey of 53 RDEB patients and their caregivers, dressing change frequency depended on whether the wound was infected [53]. For non-infected wounds 42% (22/53) changed dressings daily and 34% (18/53) changed dressings every other day; for infected wounds, 47% (25/53) changed dressings daily, 13% (7/53) changed dressings every other day, and 11% (6/53) changed dressings two to three times per day.

##### Duration of dressing changes

The time required for wound care was considerable (Table 7) [53, 59, 79]. A single-center survey of patients (N = 11) in the UK reported a median time of 25.25 h per week (101 h per month) spent on wound dressings [76]. In a US and Canadian survey, the majority of patients (55/90, 61%) required the assistance of one person for dressing changes; 17% (15/90) required two assistants [79]. Only 22% (20/90) did not require assistance.

**Table 7:** Time required for a dressing change and/or wound care in patients with RDEB

	Bruckner [53]	Jeon [59]	Shayegan [79]
N, patients with RDEB	53	13	90
Definition	Whole body wound care including preparation and cleanup	Dressing change	Dressing change
Time required for dressing change, n (%)			
< 2 h	19 (36)	11 (85)	NR
2–3 h	21 (40)	2 (15)	27 (30)^a^
> 4 h	13 (25)	0 (0)	NR

NR, not reported; RDEB, recessive dystrophic epidermolysis bullosa

^a^2 hours was most commonly reported time taken to change dressings; no other time data on patients with RDEB were reported

#### Overall financial burden

The overall financial burden of RDEB was reported in two studies [59, 61]. In the Korean survey, over half of respondents (7/13, 54%) reported always experiencing economic burden due to dressing materials [59]. Similarly, in a US survey, half (16/32, 50%) of respondents reported a high or severe level of impact on finances due to their disease [61].

Data on the indirect costs of RDEB, including impact on employment and productivity loss in patients with RDEB and their families, were not identified.

## Discussion

This study is, to our knowledge, the first published systematic literature review (SLR) to comprehensively describe the clinical, humanistic, and economic burden of disease in patients with RDEB. A total of 65 studies met inclusion criteria for this systematic literature review, and, together, the data indicated that the cost of disease care, including wound management, in patients with RDEB and their families is considerable. Significant time was spent dressing wounds and the patient-borne expenses associated with wound dressing materials were high.

Patients with RDEB experienced a significant impact on QOL due to their disease compared to other EB subtypes, skin diseases, and healthy controls and experience severe limitations in function and social activities. Furthermore, the humanistic and economic burden of RDEB extended beyond the patient to affect families and their interpersonal relationships.

A considerable burden was associated with large wounds, associated pain and itch, and multiple other comorbidities including infection, anemia, strictures and stenoses, contractures, difficulty walking, and failure to thrive. Patients with RDEB also had an increased risk of premature mortality due to pneumonia, sepsis, organ failure, and failure to thrive. Many patients developed SCC and associated complications in adulthood which can frequently be lethal. Finally, patients with RDEB underwent serious and intensive surgeries to manage their disease including esophageal dilation, gastrostomy tube placement, pseudosyndactyly release, and amputation. Our review also found that approximately 1 out of every 4 patients with RDEB do not undergo any confirmatory diagnostic testing and 1 out of every 3 do not undergo genetic analysis, though both are recommended by clinical practice guidelines [23, 80]. This is in contrast to the US and many western European countries, where the vast majority of RDEB patients are genotyped. Identification of the causative mutation via genetic testing provides patients with a definitive diagnosis, estimation of disease prognosis, and potential inclusion into clinical trials [81].

One systematic review of the natural history of RDEB reported preliminary results as the first stage of development of a longitudinal cohort study in the UK (PEBLES) [82]. The authors identified limitations such as small sample sizes, high numbers of single-center studies, limited longitudinal data, unclear or no identified RDEB subtypes, and mixing of results between RDEB and other EB subtypes. Furthermore, they identified limited-to-no data on subjective or psychosocial aspects of RDEB and the economic burden of the disease. An SLR conducted by Montaudié and colleagues on SCC and EB reported the highest incidence of SCC in EB to occur in patients with RDEB, development of SCC to arise primarily in upper and lower extremities, and in areas with chronic wounds [83]. Findings from both of these reviews are consistent with our own.

### Limitations of the existing literature

Several limitations within the existing body of literature were identified. First, sample sizes of studies were generally small and many studies were single-center, making generalizations from the study population to the larger RDEB population difficult or impossible. Additionally, almost all studies were cross-sectional, without longitudinal assessment of clinical, economic, or humanistic burden over the patient’s lifetime. The severe and lifelong nature of RDEB would best be assessed by following patients over months or years to evaluate changes over time.

Second, as there are likely RDEB patients with milder phenotypes who have been misdiagnosed [3] or were underreported, these milder RDEB presentations are likely underrepresented in the literature. Thus, it is likely that severe and systemic manifestations such as non-esophageal strictures and stenoses, pseudosyndactyly, and microstomia, are overreported. Furthermore, in some studies, there is no distinction made between RDEB subtype or severity of disease. Additionally, some studies also included other types of EB such as DDEB or EBS, making generalizations about RDEB more difficult.

Third, economic data was limited and cost comparisons across studies and populations were difficult due to differences in currencies, health systems, and cost definitions. Furthermore, indirect costs such as impact on employment and productivity loss were not available. Additionally, evidence on satisfaction and/or burden associated with treatment was minimal.

Finally, disease burden in the literature may have been applicable to RDEB patients but reported in a larger EB cohort, and thus, not met inclusion criteria into the review. For instance, Danial and colleagues highlighted itch as the most bothersome symptom among all EB patients, including those with RDEB, but these data were not included in our results as they were not specific to RDEB patients [57].

Despite these limitations, the available literature suggests that the clinical, humanistic, and economic burden of RDEB is substantial.

### Future research directions

Further research on the long-term impact of RDEB is needed to better understand how the burden of disease changes over the patient lifetime and stratified by disease severity. Recent research has shown a moderate-to-major financial impact of disease on patients with RDEB and high out-of-pocket dressing costs [84], but quantification of the economic burden across populations, geographies and healthcare systems is needed to provide appropriate care for patients. Further evaluation of the presence of anxiety, depression, and other mental health disorders that impact the humanistic burden of RDEB is also needed. Additionally, future research should measure the change in burden of disease as disease modifying treatments for RDEB, such as gene therapies, enter the market and are utilized.

## Conclusion

Collectively, the evidence identified in this review suggests a critical unmet need for RDEB treatment options that addresses the underlying disorder. RDEB is associated with significant humanistic and economic burden on patients and their families/caregivers in addition to the clinical burden. New therapies that target the underlying disorder and stand to reduce wound burden could help address the overall disease burden.

## Supplementary Information

**Additional file 1:** Included studies.

## Data Availability

Data sharing is not applicable to this article as no datasets were generated or analyzed during the current study.

## Abbreviations

BMI: Body Mass Index
C7: Type VII collagen
DDEB: Dominant dystrophic epidermolysis bullosa
DEB: Dystrophic epidermolysis bullosa
DEBRA: Dystrophic Epidermolysis Bullosa Research Association
EB: Epidermolysis bullosa
EBS: Epidermolysis bullosa simplex
iscorEB: Instrument for scoring clinical outcomes of research for epidermolysis bullosa
PROM: Patient-reported outcome measure
QOL: Quality of life
QOLEB: Quality of Life in Epidermolysis Bullosa
RDEB: Recessive dystrophic epidermolysis bullosa
SCC: Squamous cell carcinoma
SLR: Systematic literature review
UK: United Kingdom
US: United States
VAS: Visual Analogue Scale

## Footnotes

1 All costs were converted to USD based on November 5, 2020 exchange rate.
