# Supplementary material for: A systematic literature review of the disease burden in patients with recessive dystrophic epidermolysis bullosa
Source: Orphanet J Rare Dis. 2021 Apr 13;16:175. doi: 10.1186/s13023-021-01811-7 (PMC8045359; doi:10.1186/s13023-021-01811-7)
Supplement: Supplementary file 1 — Additional file 1: Included studies. [file 13023_2021_1811_MOESM1_ESM.docx]

Supplemental Table 1. Literature Review Search Terms

| Topic | Set | String |
| --- | --- | --- |
| RDEB disease search | S1 | MESH.EXACT.EXPLODE("Epidermolysis Bullosa Dystrophica") OR EMB.EXACT.EXPLODE("epidermolysis bullosa dystrophica") OR MJEMB.EXACT("epidermolysis bullosa") OR MJMESH.EXACT("Epidermolysis Bullosa") OR TI,AB(“recessive dystrophic epidermolysis bullosa” OR “RDEB-sev gen” OR “RDEB-sev” OR “RDEB”) |
| Clinical burden search | S2 | MESH.EXACT(Morbidity) OR EMB.EXACT(morbidity) OR TI,AB(“symptom burden” OR “symptoms burden” OR “burden of symptoms” OR morbidity OR "clinical burden" OR "treatment burden" OR "disease burden" OR sequelae OR sequela OR consequence OR consequences OR complication OR complications OR dysfunction OR dysfunctions OR dysfunctional OR impairment OR impairments OR deficit OR deficits OR disability OR disabilities) |
| Humanistic burden search | S3 | EMB.EXACT(“International Classification of Functioning, Disability and Health” OR “quality of life” OR “short form 36” OR “patient-reported outcome” OR “patient preference” OR “questionnaire” OR “quality adjusted life years”) OR EMB.EXACT.EXPLODE(“health status indicator”) OR MESH.EXACT(“International Classification of Functioning, Disability and Health” OR “Quality of Life” OR “Value of Life” OR “Patient Reported Outcome Measures” OR “Patient Preference” OR Questionnaires OR “Quality-Adjusted Life Years”) OR MESH.EXACT.EXPLODE(“Health Status Indicators”) OR TI,AB(burden OR (impact NEAR/3 (caregiver OR family OR families OR society OR societal or patient OR person)) OR “unmet need” OR “disability adjusted” OR “disability-adjusted” OR DALY* OR “dartmouth coop” OR “Duke health profile” OR EQ5D OR “EQ 5D” OR EQ OR EUROQOL OR “EURO QOL” OR EUROQUAL OR “EURO QUAL” OR ((daily OR day) NEAR/3 activit*) OR “functional status” OR FSQ OR (function* NEAR/5 (reduc* OR impair* OR decrease* OR impact*)) OR “quality of life” OR hrqol OR hrql OR hql OR hqol OR “hr qol” OR “h qol” OR “health stat*” OR “life quality” OR (health NEAR/3 (status OR indicator*)) OR “Nottingham health” OR NHP OR PQOL OR “perceived quality” OR QLS OR “quality of life scale” OR wellbeing OR “well being” OR “well-being” OR QWB OR rosser OR SF OR “short form” OR “short-form*” OR “shortform*” OR SF6 OR SF8 OR SF12 OR SF20 OR SF36 OR “sickness impact” OR SIP OR “patient reported” OR “patient-reported” OR “self reported” OR “self-reported” OR QALY* OR QALD* OR QALE* OR QTIME* OR (utilit* NEAR/3 (valu* OR measur* OR health OR life OR estimat* OR elicit* OR disease OR score* OR weight OR instrument OR instruments OR index)) OR “quality adjusted” OR “quality-adjusted” OR “life year*” OR “life-year*” OR “health year*” OR “health-year*” OR disutilit* OR “willingness to pay” OR “willingness-to-pay” OR WTP OR (preference* NEAR/3 (valu* OR measur* OR health OR life OR estimat* OR elicit* OR disease OR score* OR instrument OR instruments OR index)) OR “healthy utility index” OR hui OR hui1 OR hui2 OR hui3 OR “standard gamble” OR “time trade off” OR “time tradeoff” OR TTO OR QOL OR “health assessment questionnaire” OR “health assessment questionnaires” OR HAQ) |
| Economic burden search | S4 | MESH.EXACT(“Cost of Illness” OR “Economics, Hospital” OR “Economics, Nursing” OR “Economics, Pharmaceutical” OR “Fees and Charges” OR “Economics, Dental” OR “Employer Health Costs” OR “Efficiency” OR “Presenteeism” OR “Absenteeism”) OR MESH.EXACT.EXPLODE(“Health Care Costs” OR “Health Expenditures” OR “Economics, Medical” OR “Salaries and Fringe Benefits”) OR EMB.EXACT(“cost of illness” OR “drug cost” OR “productivity” OR “medical leave” OR “presenteeism” OR “absenteeism”) OR EMB.EXACT.EXPLODE(“health care cost” OR “salary and fringe benefit”) OR TI,AB(presenteeism OR absenteeism OR (cost* NEAR/3 (medical OR direct OR indirect OR drug OR pharmaceutical OR hospital OR emergency OR outpatient OR inpatient OR ambulatory OR “primary care” OR practitioner OR device OR informal OR economic OR societal OR intangible OR caregiver OR physician OR specialist OR healthcare OR “health care” OR annual* OR clinic)) OR ((burden OR impact) NEAR/3 (cost OR costs OR economic OR economics OR caregiver OR caregivers OR family OR families OR society OR societal OR employee OR employer)) OR (los* AND work AND day*) OR “sick day” OR “sick leave” OR “sickness absence” OR “work absence” OR “work incapacity” OR “work leave” OR “disability absence” OR ((resource OR healthcare OR “health care”) NEAR/5 (use OR utilization OR utilisation)) OR ((visit* OR admission* OR readmission* OR re-admission* OR stay* OR day*) NEAR/3 (physician OR emergency OR specialist OR outpatient OR inpatient OR “primary care” OR practitioner OR hospital OR clinic)) OR hospitalization OR hospitalisation OR “length of stay” OR “LOS”) |
| Publication types not of interest | S5 | (EMB.EXACT(editorial OR “case report” OR letter OR note) OR DTYPE(“Editorial” OR “Comment” OR “Letter” OR “Case Reports” OR “News” OR “Newspaper Article”) OR TI,AB(“case study” OR “case report”)) |
| Clinical burden combined | S6 | S1 AND S2 NOT S5 |
| Humanistic burden combined | S7 | S1 AND S3 NOT S5 |
| Economic burden combined | S8 | S1 AND S4 NOT S5 |
| Combined search | S9 | S6 OR S7 OR S8 |

Supplemental Table 2. Overview of Studies Included in Systematic Literature Review

| Citation | Country | Study design | Data source | Sample size | Adult/ pediatric population | Mean/  median age, years | RDEB Subtype, % | Outcomes reported |  |
| --- | --- | --- | --- | --- | --- | --- | --- | --- | --- |
| Abahussein, 1993(16) | Saudi Arabia | Single center | College of Medicine and Medical Services, King Faisal University | 10 | Pediatric | NR | NR | Symptoms |  |
| Alband, 2016(17) | UK | Registry | UK National EB Registry | 79 | Pediatric | Median, 7 | NR | Symptoms, premature mortality, SCC, procedures |  |
| Andreoli, 2002(67) | Italy | NR | NR | 11 | Both | NR | Severe, 36.4  Other, 63.6 | PROMs |  |
| Bruckner 2020(53) | US | Survey | DEBRA, EB Research Partnership | Patients, 19  Caregivers, 34 | Both | Median, 7 (caregiver-reported); 30 (patient-reported) | NR | Non-direct medical costs, PROMs, symptoms, SCC, |  |
| Bruckner, 2018(68) | US, Canada | Multicenter | Children's Hospital Colorado, Hospital for Sick Children (Toronto) | 16 | Both | NR | Intermediate, 43.8  Severe, 56.3 | Non-direct medical costs |  |
| Castelo, 2019(64) | Spain | Single center | La Paz University Hospital | 14 | Both | NR | NR | SCC |  |
| Castro, 2016(18) | NR | NR | NR | 8 | Pediatric | NR | NR | Symptoms |  |
| Cestari, 2016(69) | Brazil | Single center | Hospital de Clinicas de Porto Alegre | 19 | Both | NR | NR | PROMs |  |
| Chernyshov, 2019(19) | Ukraine, Romania | NA | NR | 12 | Pediatric | Mean, 2.9  Median, 3 | Moderate, 41.7 Severe, 58.3 | PROMS, symptoms |  |
| Choi, 2017(61) | NR | Registry | AltaVoice | 32 | Both | Mean, 23 | NR | Financial burden, PROMs, symptoms |  |
| Colomb, 2012(20) | France | Single center | French Reference Centre of Genetic Skin disorders | 12 | Pediatric | Mean, 9 | Severe, 100 | Symptoms, procedures |  |
| Čolović, 2017(21) | Serbia | Single center | Clinic for Pediatric and Preventive Dentistry, the University of Belgrade. | 12 | Pediatric | NR | NR | Symptoms, procedures |  |
| Danial 2015(57) | North America | Multicenter | 7 EBCRC centers | 77 | Both | NR | NR | PROMs, symptoms |  |
| Eismann, 2014(70) | US | Single center | Cincinnati Children's Hospital Medical Center | 32 | Pediatric | NR | NR | PROMs |  |
| Eng 2020(54) | Global | Registry | EBCare Registry | 85 | Both | Mean, 21 | Intermediate, 17.6  Severe, 25.9 | PROMs, symptoms, procedures |  |
| Fantauzzi, 2008(22) | Brazil | NR | NR | 51 | Both | NR | NR | Symptoms |  |
| Feinstein, 2018(23) | US, Canada | Registry | Epidermolysis Bullosa Clinical Characterization and Outcomes Database | 283 | Both | NR | Non-severe, 39.2  Severe, 38.5  Unknown, 22.3 | Symptoms, Procedures |  |
| Feldmann, 2012(24) | Germany | NR | NR | 13 | Pediatric | NR | Severe, 69.2  Other, 30.8 | PROMs, symptoms |  |
| Fine, 2004(58) | US | Registry | National EB Registry^a^ | 80 | Both | NR | Intermediate, 60.0  Severe, 40.0 | Direct costs and HRU, PROMs, symptoms |  |
| Fine, 2004(27) | US | Registry | National EB Registry^a^ | 421 | Both | NR | Intermediate, 63.2  Severe, 32.8  Inversa, 4.0 | Symptoms, procedures |  |
| Fine, 2005(28) | US | Registry | National EB Registry^a^ | 425 | Both | NR | Intermediate, 62.6  Severe, 33.4  Inversa, 4.0 | Symptoms, procedures |  |
| Fine, 2005(75) | US | Registry | National EB Registry^a^ | 80 | Both | NR | Intermediate, 60.0  Severe, 40.0 | Symptoms, Impact on families and caregivers |  |
| Fine, 2007(29) | US | Registry | National EB Registry^a^ | 425 | Both | Mean, 19.2 | Intermediate, 62.8  Severe, 33.2  Inversa, 4.0 | Symptoms |  |
| Fine, 2008(25) | US | Registry | National EB Registry^a^ | 407 | Both | NR | Intermediate, 65.6  Severe, 34.4 | Symptoms |  |
| Fine, 2008(63) | US | Registry | National EB Registry^a^ | NR | Both | NR | NR | Premature mortality, SCC |  |
| Fine, 2008(30) | US | Registry | National EB Registry^a^ | 424 | Both | NR | Intermediate, 62.7  Severe, 33.3  Inversa, 4.0 | Symptoms, procedures |  |
| Fine, 2009(26) | US | Registry | National EB Registry^a^ | 421 | Both | NR | Intermediate, 62.5  Severe, 33.5  Inversa, 4.0 | Symptoms, SCC, procedures |  |
| Flannery 2020(78) | Ireland | NR | NR | 5 | Pediatric | Mean, 7.3  Median, 8 | NR | Direct costs and HRU |  |
| Freeman, 2008(31) | UK | Single center | Great Ormond Street Hospital for Children | 57 | Pediatric | NR | NR | Symptoms, procedures |  |
| Frew, 2009(71) | Australia | NR | NR | 16 | NR | NR | NR | PROMs |  |
| Grocott, 2013(76) | UK | Single center | UK EB specialist center | 11 | Adults | Mean, 27.5  Median, 28 | Severe, 81.8  Inversa, 9.1  Pruriginosa, 9.1 | Direct costs and HRU, non-direct medical costs |  |
| Guerra-Leal, 2016(32) | Mexico | Registry | EB research and support foundation | 14 | Both | Mean, 26.3  Median, 26.5 | Severe, 35.7  Other, 64.3 | Symptoms |  |
| Haynes, 2012(65) | UK | Single center | Departments of Dietetics and Epidermolysis Bullosa, Hospital for Children, London | 15 | Pediatric | NR | Severe, 100 | Procedures |  |
| Horn, 2002(33) | Scotland | Registry | UK epidermolysis bullosa registry | 14 | NR | NR | Non-severe, 28.6  Severe, 57.1  Inversa, 14.3 | Symptoms |  |
| Hwang, 2015(34) | Australia | Registry | Australasian Epidermolysis Bullosa Registry | 25 | Both | NR | NR | Symptoms, procedures |  |
| Intong, 2017(35) | Australia, New Zealand | Single center,  Registry | Royal Australian New Zealand College of Obstetricians and Gynecologists, Australasian EB registry | 16 | Pediatric | NR | NR | Symptoms, procedures |  |
| Jefferies, 2013(36) | US | Single center | Cincinnati Children's Hospital Medical Center | 45 | NR | NR | NR | Symptoms |  |
| Jeon, 2016(59) | South Korea | Single center | Department of Dermatology, Gangnam Severance Hospital | 13 | Both | Mean, 21.57 | Intermediate, 46.2  Severe, 53.8 | Direct medical costs and HRU, non-direct medical costs, financial burden, PROMs, symptoms |  |
| Jones, 2016(37) | UK | Single center | Departments of Ophthalmology and Dermatology, Great Ormond Street Hospital for Children, | 36 | Pediatric | NR | NR | Symptoms |  |
| Kaneko, 2000(38) | Japan | Single center | Juntendo University School of Medicine | 11 | Both | Mean, 17.7  Median, 18 | Generalized, 81.8  Localized, 18.2 | Symptoms |  |
| Kim, 2018(6) | Australia, New Zealand | Registry | Australasian EB Registry | 16 | NR | NR | Intermediate, 54.1  Severe, 43.7 | Symptoms, SCC, procedures |  |
| Kirkorian, 2014(77) | US | Costing model | NA | NA | Pediatric | NA | NA | Direct medical costs and HRU |  |
| Lara-Corrales, 2010(39) | Australia | Multicenter | Physicians participating in ‘‘ebcarenetwork,’’ | 15 | Both | Mean, 12.4 | NR | Symptoms, procedures |  |
| Lin, 2006(66) | US | Single center | University-affiliated pediatric hospital | 25 | Pediatric | Mean, 7.6 | NR | Procedures |  |
| Markos, 2016(40) | Croatia | Single center | Endoscopy Unit, Department of Gastroenterology and Hepatology, University Hospital Center Zagreb | 6 | Both | Mean, 20.3 | NR | Symptoms, procedures |  |
| McDonald, 2018(41) | UK | Single center | Pediatric interventional radiology center | 71 | Pediatric | NR | Intermediate, 7.0  Severe, 88.7  Inversa, 4.2 | Symptoms, procedures |  |
| Mellado, 2018(42) | Chile | Registry | Dystrophic Epidermolysis Bullosa Research Association, Chile | 31 | Both | Mean, 17.0 | NR | Symptoms, procedures |  |
| Mellerio, 2016(62) | NA | NA | NA | 54 | Both | NR | NR | Direct costs and HRU, symptoms |  |
| Moss, 2009(74) | UK | Registry | National Health Service-funded EB Service | 34 | Both | Median (severe subtype), 3.7  Median (other subtype), 11.1 | Severe, 67.6  Other, 32.4 | PROMs |  |
| Nomura, 1993(43) | Japan | Single center and medical literature | Hirosaki University School of Medicine | 28 | NR | NR | NR | Symptoms |  |
| Rao, 2011(44) | UK | Single center | A pediatric EB center | 18 | Pediatric | Median, 12.42 | NR | Symptoms |  |
| Reimer 2019(55) | Germany | Single center | Main German referral center | 157 | Both | Median (generalized subtype), 5.6 Median (severe subtype), 10.4 | Intermediate, 48.4  Severe, 51.6 | Symptoms, procedures |  |
| Ryan, 2015(46) | US | Single center | NR | 45 | NR | NR | NR | Symptoms |  |
| Ryan, 2016(45) | US | Single center | NR | 45 | Both | Mean, 12.5  Median, 11 | NR | Symptoms |  |
| Sampogna, 2013(47) | Italy | Registry | Instituto Dermopatico dell’Immacolata IRCCS | 62 | Both | Mean, 19.2 | NR | PROMs, impact on families and caregivers, symptoms |  |
| Schräder, 2018(60) | Netherlands | Single center | Center for Blistering Diseases at the Department of Dermatology, University Medical Center Groningen | 5 | NR | NR | NR | PROMs, symptoms |  |
| Schwieger-Briel, 2015(72) | Canada | Registry and single center | EB Care Network; patients attending an EB clinic | 9 | Both | NR | NR | PROMs |  |
| Serrano-Martínez, 2003(48) | Spain | Multicenter | NR | 35 | Both | Mean, 16.7 | Generalized, 100 | Symptoms |  |
| Shayegan 2020(79) | US, Canada | Registry | EBCCOD | 93 | Both | NR | NR | Non-direct medical costs |  |
| Sidwell, 2000(49) | UK | Single center | Department of Pediatric Dermatology, Great Ormond Street Hospital for Children | 6 | Pediatric | NR | Severe, 100 | Symptoms |  |
| Soon 2020(73) | UK | Single center | National Center of Excellence | 18 | Pediatric | NR | NR | PROMs |  |
| Stellingsma, 2011(50) | Netherlands | Single center | Department of Dermatology, University Medical Centre Groningen | 10 | both | Median, 10.8 | Severe, 40.0  Inversa, 50.0  Other, 10.0 | Symptoms |  |
| Teng 2019(56) | US | Single center | Stanford EB clinic | 40 | Both | NR | NR | Symptoms |  |
| Tong, 1999(51) | UK | Single center | Great Ormond Street Children’s Hospital | 72 | NR | NR | NR | Symptoms, procedures |  |
| Wagner, 2010(52) | US | Single center | EB clinical trial | 6 | Pediatric | Mean, 6.8 | NR | Symptoms |  |
| Abbreviations: EB, epidermolysis bullosa; EBCCOD, EB Clinical Characterization and Outcomes Database; HRU, healthcare resource utilization; NA, not applicable; NR, not reported; PROM, patient-reported outcome measure; SCC, squamous cell carcinoma; UK, United Kingdom; US, United States  ^a^ Sample sizes vary due to available data | | | | | | | | | |
